# How breast cancer treatments affect the quality of life of women with non-metastatic breast cancer one year after surgical treatment: a cross-sectional study in Greece

**DOI:** 10.1186/s12893-020-00871-z

**Published:** 2020-09-21

**Authors:** Aris Yfantis, Pavlos Sarafis, Ioannis Moisoglou, Maria Tolia, George Intas, Ioanna Tiniakou, Konstantinos Zografos, George Zografos, Marianna Constantinou, Athanasios Nikolentzos, Michalis Kontos

**Affiliations:** 1 General Public Hospital of Lamia, Papasiopoulou Street, 35100 Lamia, Greece; 2 Department of Nursing, Cyprus University of Technology, 30 Archbishop Street, 3036 Limassol, Cyprus; 3 University Hospital of Crete, Voutes, 71110 Heraklion, Crete Greece; 4 General Public Hospital Nikaia “Agios Panteleimon”, D. Mantouvalou 3, Nikaia, 18454 Athens, Greece; 5 General Public Hospital “Laiko”, School of Medicine, National and Kapodistrian University of Athens, Agiou Thoma 17, 11527 Athens, Greece; 6 General Public Hospital “Hippocration” Hospital, School of Medicine, National and Kapodistrian University of Athens, Vasilissis Sofias Ave. 114, 11527 Athens, Greece; 7 School of Social Sciences, Hellenic Open University, Parodos Aristotelous 18, TK 26335 Patras, Greece

**Keywords:** Adjuvant therapy, Breast cancer, Care, Quality of life, Surgery

## Abstract

**Background:**

The continuously increasing survivorship of female breast cancer makes the monitoring and improvement of patients’ quality of life ever so important. While globally there is a growing body of research on health-related quality of life 1 year after surgical treatment for non-metastatic breast cancer, up-to-date information regarding Greek patients is scarce.

**Objective:**

To measure the level of QoL of non-metastatic BC survivors in Greece 1 year after surgery.

**Methods:**

A sample of 200 female breast cancer survivors aged 18 to 75, who followed up as outpatients in five public hospitals were included in this cross-sectional study. All recruited patients agreed to participate in the study (100% response rate). Quality of life data were collected through the EORTC QLQ-C30 as well as BR23 questionnaires.

**Results:**

Cronbach’s alpha for all scales of the two questionnaires was from 0.551 to 0.936 indicating very good reliability. According to the Multiple Linear Regression, older patients showed a lower future perspective (*p* = .031), with those living in rural areas, which was associated with more financial difficulties (*p* = .001). Women with tertiary education and those who had been hospitalized in a university hospital recorded better on global health status (*p* = .003 and .000 respectively). Patients who underwent chemotherapy reported better scores in the emotional function sub-scale (*p* = .025). Women with reconstruction and at least one complication appeared to have significantly better scores in future perspective and social function (*p* = .005, .002 respectively).

**Conclusions:**

Breast cancer survivors were found to have an overall good quality of life, functioning/symptoms scores and were satisfied with the provided care.

## Background

BC (breast cancer) has a high incidence, with approximately 5000 new cases reported each year in Greece and 1.67 million cases reported worldwide [1]. Because of the advances of systemic therapies, the widely used screening programs, and the high incidence of BC, the number of BC survivors is continuously increasing [2]. However, despite their benefits these therapies have numerous side effects, which adversely affect patients’ quality of life (QoL). Cardiovascular complications, infections, implant removal in patients with implant reconstruction, higher adjusted total cost and complication-related cost compose some of the most important complications and side effects of the treatments [3–5].

The assessment of the health-related quality of life (HRQoL) is a reliable tool which measures the degree to which a chronic disease or condition and its treatment can affect patients’ QoL. It provides healthcare professionals with data about health status and highlights problems that are difficult to be recognized during daily care or consultation [6]. The dimensions that compose QoL include physical, emotional, social, cognitive, and sexual function [7]. Even after 5 years of treatment, QoL continues to impair a large proportion of patients. Long-term problems include cognitive function, sleep disorders, sexual issues, hot flashes, pain, fatigue, and polyneuropathy [8].

These QoL problems should not be underestimated, because they are prognostic factors for further serious problems. More specifically, depression and physical disability are associated with suicidal ideation of BC survivors [9]. Moreover, the frequency of psychological disorders is correlated with sexual ones, which means that the BC survivors are experiencing a vicious circle of problems, where one problem gives positive feedback to the other [10].

Regarding cognitive impairment, including the domains of memory, speed of processing, and concentration, BC survivors reported this impairment as detrimental to their self-confidence and social relationships [11]. Also, survivors that are functionally deteriorated are more likely to experience adverse economic situations, such as changes in earnings, delayed work return, and problems with insurance coverage [12]. Undoubtedly, in BC treatment, the patients’ survival is the clinicians’ first and most important goal. However, the disease and its treatments have some side effects that affect negatively BC survivors’ life; the patient’s QoL, which is often overlooked, is impaired.

### Theoretical framework

The present study, guided by the conceptual framework proposed by Ware [13] and Fayers et al. [14], proposed four concepts of QoL that must be explored during the studies of cancer and other diseases. The concepts consist of personal functioning, psychological distress/well-being, general health perceptions, and social/role functioning. Personal functioning is defined as the performance of tasks or the capacity to perform tasks that most people do daily, including self-care, mobility, and physical activities. The psychological distress/well-being is referred to as a personal mental health status. General health perceptions constitute a self-rating of health, which encompasses patients’ evaluation of personal health according to the three preceding concepts (disease status, personal functioning, and mental health). The role refers to the patients’ performance or the capacity to perform activities associated with patients’ usual role, including employment or homemaking and it reflects both on a person’s health status and the demands of that person’s chosen role activity. However, the effect of the disease’s symptoms should also be measured, as they can affect patients’ QoL [13]. According to Fayers, disease symptoms such as vomiting, pain, and diarrhea, called causal indicators, can cause impairment of QoL [14].

There is evidence that 1 year after surgical treatment for non-metastatic BC, HRQoL shows significant improvement [15, 16]. In Greece, there are only a few studies regarding the HRQoL on BC survivors [17, 18]. More particularly, the first study was limited to stage II BC patients, while the second study included women who were still undergoing treatment. The present study had the largest BC Greek sample compared to previous studies in Greece and attempts to investigate the potential effects of variables that haven’t been studied yet (residence, type of hospital, complications).

Thus, the study had two aims: (a) to explore the effects of cancer treatments on the QoL of non-metastatic BC survivors in Greece 1 year after surgery and (b) to investigate the association of treatment-related side effects and patients’ demographic and clinical characteristics with QoL.

## Methods

### Study design

The study protocol was approved by the Ethics Committee of the “Hippocratio” General Hospital of Athens (reference number 15564/ 29.8.2014) and then it was approved by the Ethical Committees of the participant hospitals.

This cross-sectional descriptive study was conducted from December 1st, 2014 to January 31st, 2017 and was implemented in five high volume public hospitals in three distinct regions in Greece, in order to include patients from rural, urban, and the Athens metropolitan area (AMA). It is worth noting that the AMA is a heavily urbanized area with high quality medical services. For this reason, the variable “place of residence” includes three categories (rural areas, urban areas, AMA), so as to receive useful information about the inequalities in meeting survivors’ needs and the provision of quality health services.

### Participants

A convenience sample of 200 Greek BC survivors, who had undergone surgery the year before, had completed BC treatments (except hormonal therapy) and had already returned to their daily routine, was constructed in order to participate in the study. We opted for patients who had undergone surgery a year ago so that they would have completed their treatments and returned to their daily routine. The participants were followed-up as outpatients during their routine visits to the surgery clinic, where the researchers contacted them and collected their demographic data 1 day before their surgery. Inclusion criteria for the participants were defined as: i) age 18 to 75, ii) ability to understand, consent, and complete the questionnaire, iii) surgical treatment for BC approximately 1 year earlier, iv) outpatients in public hospitals. Exclusion criteria were: i) history of other malignancies, ii) metastatic cancer, iii) inability to communicate in Greek, iv) incomplete BC treatment. All recruited women consented to participate in the study (100% response rate).

The participants’ mean age was 56.2 years (SD = 12.6). There were 36 (18%) patients who underwent breast-conserving surgery, 97 (48.5%) women who underwent mastectomy without reconstruction and 67 (33.5%) with reconstruction. Within the sample, 77% of the patients underwent chemotherapy, 65% received hormonal therapy and 44.5% radiotherapy. Most of the women had axillary node clearance (78%). Complications appeared in 3.5% of the sample (Table 1).

**Table 1:** Demographic and clinical characteristics of the study participants (*N* = 200)

	Ν	N %
**Residence**		
Athens Metropolitan Area	46	25.7%
Urban center	78	43.6%
Rural area	55	30.7%
**Education level**		
Primary	60	30.0%
Secondary	23	11.5%
High school	62	31.0%
Tertiary	35	17.5%
No education	20	10.0%
**Type of hospital**		
General Hospital	103	51.5%
University Hospital	97	48.5%
**Type of surgery**		
Breast conserving surgery	36	18.0%
Mastectomy without reconstruction	97	48.5%
Mastectomy and reconstruction	67	33.5%
**Type of axillary therapy**		
Sentinel lymph node biopsy	44	22.0%
Axillary node clearance	156	78.0%
**Chemotherapy**		
Yes	154	77.0%
No	46	23.0%
**Radiotherapy**		
Yes	89	44.5%
No	111	55.5%
**Hormonal therapy**		
Yes	130	65.0%
No	70	35.0%
**Complications**		
Yes	7	3.5%
No	193	96.5%

### Instruments

The Greek version of the EORTC-QLQ-C30 and BR23 questionnaires was used [19, 20]. The QLQ-C30 questionnaire consists of 30 items and assesses the QoL of patients. It is a general questionnaire that is not specific for BC and includes five functional scales, physical, cognitive, emotional, social function and role. It also consists of symptom scales (fatigue, nausea and vomiting, pain, dyspnea, insomnia, appetite loss, constipation, diarrhea, and financial difficulty) and global health status / QoL scales (GHS). Most questions are answered on a 4-point Likert scale (not at all to very much) and participants could rate each question on a scale of 0 to 100. Higher scores for a functional scale indicate a high/healthy level of functioning, a higher score for the GHS indicates a high QoL and a higher score for a symptom scale/items represents a high level of symptomatology/problems [21].

The QLQ-BR23 questionnaire consists of 23 items and assesses the QoL of BC patients. It is designed specifically for BC and includes four functional scales: body image, sexual functioning, sexual enjoyment, and future perspective and a “symptom” scale that describes the side effects of the treatment, breast and arm symptoms and hair loss. All items were rated on a 4-point Likert scale (not at all to very much). The scoring approach for the QLQ-BR23 is identical to the principle we used for the function and symptom scales/single items of the QLQ-C30 [22].

The demographic and clinical characteristics of the participants, which included the age, the place of residence, the educational level, the type of hospital, the type of therapy and the complications were also collected.

### Statistical methods

For the descriptive statistical analysis, continuous variables were expressed as mean and standard deviation (SD), while the discrete variables were expressed as frequency and relative frequency (%). The internal consistency coefficient was studied using the Cronbach’s Alpha coefficient of the two questionnaire scales with at least 2 items to measure the reliability. Values greater than or closer to 0.7 are considered acceptable. Values between 0.5 and 0.6 are considered sufficient in the initial stages of a study. If the alpha value exceeds 80%, then it is considered a particularly reliable analysis.

Multiple linear regression was used to investigate the relationship between the sub-scales of the two questionnaires with demographic factors. The Forward method, which is used in exploratory studies where there is no prior knowledge of the independent variables that affect the dependent variables, was applied to investigate the independent variables. Statistical analysis of the data was performed using SPSS-25 statistical software. The minimum value of the level of statistical significance, *p*-value, in all statistical tests was set at 0.05.

## Results

According to the baseline statistics of the two questionnaires (Table 2), the overall composite score of QLQ-C30 functional scales indicated a fairly good QoL with low-intensity symptomatology. A moderate degree of patient functionality and fewer symptoms were reported in the QLQ-BR23. Cronbach’s alpha coefficient showed that the questionnaires were overall considered to be of high quality in terms of reliability. The only exception was observed in the physical function sub-scale (0.551).

**Table 2:** Statistical indicators of the scores of all subscales of the “QLQ-C30/BR23” questionnaires

Scales and sub-scales	Items	Mean	SD	Cronbach’s Alpha
EORTC - QLQ-C30				
Physical Function	5	81.2	16.5	0.551
Role Function	2	71.0	25.8	0.848
Emotional Function	4	70.0	27.0	0.851
Cognitive Function	2	86.3	19.7	0.704
Social Function	2	80.7	25.0	0.824
**Overall composite score** (functional scales)		77.8	16.3	
Global Health Status	2	55.4	25.0	0.936
Fatigue	3	34.1	23.6	0.855
Nausea and Vomiting	2	8.3	19.4	0.905
Pain	2	20.9	22.5	0.829
Dyspnea		21.4	26.1	
Insomnia		26.9	31.8	
Appetite Loss		13.2	25.9	
Constipation		15.2	26.9	
Diarrhea		5.9	17.5	
Financial Difficulties		24.3	32.5	
**Overall composite score** (symptom scales)		19.0	17.7	
EORTC - QLQ-BR23				
Body Image	4	73.7	28.8	0.892
Sexual Function	2	18.6	24.0	0.896
Sexual Enjoyment		43.8	20.6	
Future Perspective		56.1	34.7	
**Overall composite score** (functional scales)		48.0	12.2	
Systemic Therapy Side-Effects	7	24.5	20.5	0.819
Breast Symptoms	4	13.6	20.7	0.872
Arm Symptoms	3	16.1	19.5	0 .812
Upset by Hair Loss		35.3	25.5	
**Overall composite score** (symptom scales)		22.4	14.6	

Multiple linear regression analysis was used to investigate the relationship between the sub-scales of the two questionnaires with independent factors (Table 3). According to our findings, the variable “age” was an independent factor for future perspective, breast/arm symptoms and pain, with older patients having a lower future perspective, less breast/arm symptoms and pain. Furthermore, the variable “residence” showed that women with BC who lived in rural places had more financial difficulties and a low score on body image compared to women who lived urban areas and the AMA. On the contrary, urban women appear to score better on emotional function, they do not have severe insomnia problems, and experience fewer systemic therapy side-effects compared to women of the other two groups. High scores on the subscale of pain, diarrhea, and breast/arm morbidity were statistically significant in women residing in the AMA, who scored low on cognitive as well.

**Table 3:** Summary of Multiple Linear Regression analysis for independent variables predicting sub-scales scores of “QLQ-C30/BR23” questionnaires

	Unstandardized Coefficients				***R***^**2**^ (%)
	B	Std. Error	t	Sig.	
**Global Health Status**					**37.3**
(Constant)	−8.897	6.750	−1.318	.189	
University Hospital	25.483	4.445	5.733	.000	
Mastectomy without reconstruction	−12.415	3.223	−3.852	.000	
Tertiary education	−13.005	4.385	−2.966	.003	
Hormonal Therapy	7.742	3.146	2.461	.015	
Chemotherapy	9.103	3.603	2.526	.012	
**Physical function**					**5.9**
(Constant)	82.505	1.266	65.150	.000	
No education	−16.351	4.686	−3.489	.001	
**Role function**					**6.3**
(Constant)	76.382	2.275	33.572	.000	
Mastectomy and reconstruction	−14.235	3.952	−3.602	.000	
**Emotional function**					**7.1**
(Constant)	66.408	9.156	7.253	.000	
Radiotherapy	−7.687	4.006	−1.919	.057	
Urban center	9.756	4.056	2.405	.017	
Chemotherapy	10.683	4.727	2.260	.025	
Secondary school	−12.648	6.323	−2.000	.047	
**Cognitive function**					**7.7**
(Constant)	86.346	2.492	34.648	.000	
Athens Metropolitan Area	−7.729	3.535	−2.186	.030	
No education	−15.512	5.636	−2.752	.007	
Mastectomy without reconstruction	6.596	3.121	2.113	.036	
**Social function**					**10.3**
(Constant)	16.910	18.818	.899	.370	
Mastectomy without reconstruction	12.125	3.426	3.540	.001	
Complications	29.805	9.491	3.140	.002	
**Fatigue**					**1.8**
(Constant)	47.681	7.293	6.538	.000	
Sentinel Lymph Node Biopsy	−8.160	3.999	−2.041	.043	
**Pain**					**11**
(Constant)	26.459	9.123	2.900	.004	
Age	−.388	.130	−2.988	.003	
Radiotherapy	8.662	3.228	2.683	.008	
Athens Metropolitan Area	9.479	3.728	2.542	.012	
**Dyspnea**					**2.7**
(Constant)	40.054	8.160	4.909	.000	
Sentinel Lymph Node Biopsy	−10.887	4.474	−2.433	.016	
**Insomnia**					**2.4**
(Constant)	30.893	3.069	10.066	.000	
Urban center	−10.807	4.636	−2.331	.021	
**Appetite loss**					**5.9**
(Constant)	−7.786	5.691	−1.368	.173	
Radiotherapy	12.060	3.473	3.473	.001	
**Constipation**					**17.1**
(Constant)	−4.220	6.180	−.683	.496	
Athens Metropolitan Area	24.521	4.235	5.790	.000	
Radiotherapy	8.378	3.710	2.258	.025	
**Diarrhea**					**7.5**
(Constant)	16.586	7.164	2.315	.022	
Hormonal therapy	6.849	2.528	2.709	.007	
University Hospital	−5.744	2.378	−2.415	.017	
Sentinel Lymph Node Biopsy	−6.763	2.865	−2.360	.019	
**Financial difficulties**					**5.6**
(Constant)	18.895	2.871	6.582	.000	
Rural area	17.468	5.164	3.383	.001	
**Body image**					**27.3**
(Constant)	44.092	22.231	1.983	.049	
University Hospital	−25.415	5.139	−4.945	.000	
Complications	39.184	10.447	3.751	.000	
Mastectomy and reconstruction	−12.835	4.566	−2.811	.006	
Secondary school	−21.429	6.700	−3.198	.002	
Rural area	−11.661	4.635	−2.516	.013	
**Sexual function**					**31.6**
(Constant)	−8.897	6.750	−1.318	.189	
University hospital	25.483	4.445	5.733	.000	
Mastectomy without reconstruction	−12.415	3.223	−3.852	.000	
High school	−13.005	4.385	−2.966	.003	
**Sexual enjoyment**					**3.8**
(Constant)	48.315	2.127	22.719	.000	
Mastectomy without reconstruction	−8.489	3.008	−2.823	.005	
**Future perspective**					**9.1**
(Constant)	73.823	12.419	5.944	.000	
Mastectomy and reconstruction	15.392	5.441	2.829	.005	
Age	−.446	.205	−2.172	.031	
Secondary school	16.274	7.884	2.064	.040	
**Systemic therapy side effects**					**1.7**
(Constant)	26.359	2.027	13.007	.000	
Urban center	−6.133	3.061	−2.003	.047	
**Breast symptoms**					**9.1**
(Constant)	68.423	15.265	4.482	.000	
Athens Metropolitan Area	19.068	3.044	6.264	.000	
Complications	−22.728	7.273	−3.125	.002	
Age	−.269	.106	−2.539	.012	
**Arm symptoms**					**21.8**
(Constant)	32.808	8.400	3.905	.000	
Athens Metropolitan Area	10.801	3.280	3.293	.001	
Sentinel Lymph Node Biopsy	−7.558	3.329	−2.270	.024	
Age	−.335	.111	−3.012	.003	
Chemotherapy	9.455	3.244	2.915	.004	
No education	12.124	5.194	2.334	.021	
**Upset by hair loss**					**9.2**
(Constant)	50.282	5.536	9.082	.000	
Chemotherapy	−14.581	4.103	−3.554	.000	
High school	10.011	3.731	2.683	.008	

As far as the education level is concerned, a statistically significant relationship between education and functional scales/symptoms was revealed. Women with no education scored low on physical/cognitive function and had more arm symptoms. Additionally, patients with secondary (9 years) school educational level appeared to have a better future perspective, while they lacked emotional and body image and those with high school (12 years) education have a significantly lower score on social. Finally better GHS was reported to women with tertiary education. Regarding the type of surgery, patients who underwent mastectomy without reconstruction demonstrated higher levels of cognitive and social, but lower levels of role, GHS, sexual function and sexual enjoyment. On the other hand, women with reconstruction have significantly better scores in future perspective, but they lack in body image. In the sub-group “axillary therapy”, women who underwent sentinel lymph node biopsy showed less fatigue, dyspnea, diarrhea, and arm symptoms compared to women with axillary node clearance.

The adjuvant treatments were identified as statistically significant independent variables in QoL, as those who received hormone and chemotherapy recorded better GHS compared to women who did not receive hormone therapy and chemotherapy, while those who received radiotherapy had low scores on emotional, more pain, appetite loss and constipation compared to patients who did not receive radiotherapy. Additionally, women who received hormone therapy scored high in the subscale “diarrhea” and those who did chemotherapy scored better in the emotional sub-scale, but worse in arm symptoms. The independent variable “complications” was considered statistically significant in the subscales of social function and breast symptoms. Women who reported at least one complication had better social function and fewer breast symptoms and a lower score in body image. Finally, patients who had been hospitalized in a university hospital rated better on GHS and social function and experienced fewer diarrheas, but they lacked body image, compared to those who had been hospitalized in general hospitals.

## Discussion

According to the literature, hair loss is one of the most important side effects of chemotherapy [23]. Monfared et al. [24], in their study, found that QoL was lower in the emotional area compared to other areas. However, in our study, sample sub-scales GHS and emotional appeared to be improved in patients undergoing chemotherapy, giving a different perspective for the chemotherapy’s negative effects. This can probably be explained by the sense of security that patients have when there is a strong social support network (more common in non-urban communities), as we found a statistical correlation between rural patients and higher scores in emotional. This sense of security can also be enhanced by a specialist’s support. This is consistent with a study that showed that participation in social support groups can mitigate the adverse psychological effects of BC treatment [25].

The most common type of social support and education for BC patients is support groups [26]. Professional support and training are provided by oncology nurses and other health workers such as psychologists, psychiatrists, psychotherapists, social workers, physiotherapists [26]. Phone support is a viable option for those who cannot attend support groups or live in rural areas with limited access to cancer support services [27]. Besides, symptoms such as fatigue and nausea from BC treatment can prevent women from participating in support groups and educational meetings. Grunfeld and colleagues [28] concluded that the average cost per patient was lower in telephone than in hospital follow-up. According to previous studies, face to face interventions could be a realistic alternative to conventional cancer care. These programs through individual psychosocial support and psychological intervention are beneficial for BC patients and have a positive effect on their QoL [29].

Regarding the QLQ-BR23 questionnaire, all investigated symptoms were at fairly favorable levels in our study. The highest-scoring (worse) symptoms/items were fatigue, followed by insomnia and financial problems. Our results strengthen findings from previous studies that also report significant problems including fatigue, pain in the joints and sleeping disturbances [12, 30]. Patients living in rural areas faced more financial difficulties, compared to women in urban cities and the AMA. Economic difficulties are negatively correlated with QoL and as the functional status of the BC women impairs, more adverse economic situations appear [31]. Further research is needed to investigate the associations between urban/rural residence and QoL aspects in different environments where the concepts of urbanization and rural life may have different meanings and impacts. Such studies would help to identify whether there are disparities in survivors’ support and, if so, to enable appropriate and effective development and provision of health care and supportive care in all areas of the BC survivors.

Regarding the age, studies showed that older women with BC have significantly fewer symptoms than younger women, which is consistent with our findings [32–34]. However, contrary to our findings, an older Greek study showed that younger patients exhibited better overall QoL, fewer symptoms, and better functionality than older [18]. The difference in the results could be interpreted in the light that women who suffer the consequences of a serious illness at a relatively young age have even greater scope to redefine their priorities by addressing their lives and disease as a challenge to overcome [18]. The problems that younger women face are often very different from those encountered by older ones, such as concerns about early menopause leading to fertility loss, negative body image [33, 35, 36], sexuality, career, work and financial security [32, 36, 37].

Women with no education or basic education (≤ 12 years at school) reported lower levels in QoL sub-scales, experiencing more systemic therapy side-effects, more arm morbidity and reduced physical, cognitive, and emotional function, body image and sexual functioning. This result is in line with another Greek study that revealed a statistically significant difference between high education level and QoL [18]. Similarly, another study states that women with low education may have jobs that require more physically demanding work, which leads to delayed work return, more physical, role, cognitive function, and financial problems and suggests exercise programs for faster recovery [12].

On the other hand, a statistically significant finding revealed that patients with tertiary education reported better GHS. This can be interpreted by the different perspectives these patients adopt for their disease management, as the access to information can help them dispel prejudices [33]. Since young women are frequent users of social media and often highly educated, maybe the development of a multifunction online support hub can help them find credible and useful information [38]. In any case, if more concerns arises, they can always express it to their attending physicians [33].

Besides, the presence of fewer symptoms highlighted in our study, such as pain and breast/arm morbidity in older women may be due to their choice of surgical treatment. A recent research project with results consistent with our study concluded that younger BC women with reconstruction and more aggressive adjuvant therapies report a more negative body image than those receiving breast-conserving surgery [39]. The latter can be seen as a paradox; it can be explained, however, by the fact that young women who opt for reconstruction are more conscious of their body and have high, and often unrealistic, expectations from the breast reconstructive process [40]. The increased expectations of presumably younger women undergoing reconstruction may not be fully met regarding body image. However, they indeed restore their sexual self-confidence, which may be related to the image that the social environment shapes for them. This finding can be positively utilized by BC survivors to regain their confidence and thus their QoL after treatment.

It is also known that sexuality affects the QoL of women with BC, as those with an active sexual life report fewer QoL problems [41]. Our study suggests that women who had undergone breast reconstruction may maintain a good overall QoL in sexual function, as we found lower scores in sexual function and sexual enjoyment in those with mastectomy without reconstruction. This finding is consistent with the literature, where reconstruction had positive effects in this area of QoL [42].

Irrespective of age and the type of surgery for BC survivors, body image awareness is often associated with other issues such as social. Therefore, breast loss due to mastectomy will be perceived by many people as a serious negative factor in the recognition and sense of self as a woman. The woman feels something is missing and may feel sick and disabled. The prevailing social perception shows the female breasts as a symbol of femininity and fertility. Women characterize breast loss as a deprivation of their identity and dignity to the extent that it even affects the performance of daily activities [43]. This process is a vicious circle, as harming feminine identity can affect their overall sense of self, which can, in turn, negatively affect their body image. However, the current study showed a positive correlation between breast reconstruction and future perspective. Many BC women can accept the fact that they live in a changed body and maintain a sexual activity and pleasure with their partners, regardless of the treatments they receive, even if they had undergone a mastectomy [44].

For those who chose university hospital for their treatment, the GHS and social function were better and fewer diarrheas were reported, but they lacked body image, compared to those hospitalized in general hospitals. The comparison between the university and general hospitals has also been studied in the literature. Studies focusing on patients hospitalized in university hospitals record better quality health services, with lower mortality rates [45, 46]. Hospitals’ affiliation with a university faculty of medicine provides access to new scientific data on diagnosis, treatment and rehabilitation. Also, the existence of more specialized structures in university hospitals increases the patient’s chance of receiving better health care services.

It is worth mentioning that the Greek law dictates that all treatment decisions for BC (as well as all types of cancer) are offered by a multidisciplinary team. Breast Units are usually organized within a Department of General Surgery which employees specialized surgeons, dedicated at least partially, to breast surgery. All hospitals that participated in the study are high volume centers – including the rural ones – and, although there is no formal accreditation and no official prerequisites for Breast Units in Greece, patients involved in the study had routine full consultations regarding their treatment and its details, including systemic treatment, radiotherapy, types of surgery and reconstruction options. Treatment offered to the patients complied with the local and National guidelines and was consistent across sites.

There is scarce evidence in the literature regarding the impact of complications on QoL. In their study, Browne and colleagues found no association between BC complications and QoL after mastectomy and reconstruction [47]. In our study, women who reported complications had better social function, fewer breast symptoms and a lower score in body image. In their study, Belmonte et al. [48], found that sentinel lymph node biopsy outperforms axillary node clearance in terms of QoL, due to fewer complications from the arm, which is in accordance with the finding of the present study. However, because of the small sample size (3.5% of the sample had complications), further studies are needed to confirm and explain these findings.

### Limitations

This study was a cross-sectional and not longitudinal. Therefore, the changes in QoL over time were not captured. The sample of the study was not large and only 3.5% of the participants reported complications, so generalization of the results has to be done with great caution. Also, the period between the completion of adjuvant therapy to participation in the study has not been recorded. Therefore, patients who may have recently completed adjuvant therapy may still be under its influence and therefore report worse QoL. Finally, the study lacks in considering, according to the participants’ BC stage and their related knowledge of life expectancy, their effect on the subjects’ psychological distress and/or well-being.

## Conclusions

Advances of systemic therapies have led to increased survival rates of BC patients. Despite their important benefits, these therapies have a series of side effects, which adversely affect patients’ QoL. Measuring QoL is considered important, due to the effect it can have on the patient’s morbidity and mortality.

The present study showed that women with BC have a satisfactory level of QoL, as they scored high in functionality sub-scales. Regarding the symptoms of the disease, fatigue, insomnia, financial problems and hair loss emerged as the most important problems. Age, educational level and type of hospitalization are statistically related to the QoL. The emotional function was the least favorable; hair loss appeared to be the least desirable side-effect of the treatment. Hair is an integral part of female identity and has a negative impact on different aspects of QoL. Females with non-metastatic BC, with tertiary education, who were treated in a university hospital, resided in urban areas and had undergone sentinel lymph node biopsy reported better QoL and fewer symptoms and complications. The QoL measurement provides valuable information and reveals those factors that are crucial for patients’ health status improvement.

## Data Availability

The datasets generated and analyzed during the current study are not publicly available to preserve the privacy of the participants but are available from the corresponding author on reasonable request.

## Abbreviations

BC: Breast cancer
QoL: Quality of life
HRQoL: Health-related quality of life
AMA: Athens Metropolitan Area
EORTC: European Organisation of Research and Treatment of Cancer
GHS: Global health status
